# The Impact of Simultaneous Liver Resection for Occult Liver Metastases of Pancreatic Adenocarcinoma

**DOI:** 10.1155/2012/939350

**Published:** 2012-11-08

**Authors:** F. Klein, G. Puhl, O. Guckelberger, U. Pelzer, J. R. Pullankavumkal, S. Guel, P. Neuhaus, M. Bahra

**Affiliations:** ^1^Department of General, Visceral, and Transplantation Surgery, Charité Campus Virchow, Universitätsmedizin Berlin, Augustenburger Platz 1, 13353 Berlin, Germany; ^2^Department of Hematology and Oncology, Charité Campus Virchow, Universitätsmedizin Berlin, 13353 Berlin, Germany

## Abstract

*Backround*. Pancreas resection is the only curative treatment for pancreatic adenocarcinoma. In the event of unexpected incidental liver metastases during operative exploration patients were traditionally referred to palliative treatment arms. With continuous progress in the surgical expertise simultaneous pancreas and liver resections seem technically feasible nowadays. The aim of this study therefore was to analyze the impact of synchronous liver-directed therapy on operative outcome and overall survival in patients with hepatic metastasized pancreatic adenocarcinoma (HMPA). *Methods*. 22 patients who underwent simultaneous pancreas resection and liver-directed therapy for HMPA between January 1, 2004 and January 1, 2009 were compared to 22 patients who underwent classic pancreas resection for nonmetastasized pancreatic adenocarcinoma (NMPA) in a matched pair study design. Postoperative morbidity, preoperative, and operative data and overall survival were analyzed. *Results*. Overall survival was significantly decreased in the HMPA group. Postoperative morbidity and mortality and median operation time did not significantly differ between the groups. *Conclusion*. The results of our study showed that simultaneous pancreas resection and liver-directed therapy may safely be performed and may therefore be applied in individual patients with HMPA. However, a potential benefit of this radical surgical approach with regard to overall survival and/or quality of life remains to be proven.

## 1. Introduction

Pancreatic resection is considered the only potentially curative treatment option in patients with resectable pancreatic adenocarcinoma improving 5-year survival rates to up to 20% [1, 2]. However, pancreatic adenocarcinoma is characterized by extraordinary local tumor progression and early systemic dissemination [3]. Thus seventy-five percent of the patients with pancreatic adenocarcinoma present with locally advanced disease at the time of diagnosis resulting in reported respectability rates of only 20% [4, 5]. Therefore, despite the advanced diagnostic tools in the preoperative evaluation such as computed tomography (CT) or positron emission tomography combined with computed tomography (PET-CT) we are still repeatedly confronted with unexpected advanced tumor stages during operative exploration [6]. Especially the situation of incidental liver metastases combined with a local resectable tumor, which is a challenging subject for surgeons. The decision on whether to leave the tumor *in situ* and perform a palliative surgical bypass or to perform the intended pancreatic resection is difficult. If local pancreas resection is considered, a further question would be whether to perform the intended pancreas resection and leave the liver metastases to be treated by chemotherapy to avoid the additional operative risk of liver resection, or to undertake the intended radical resection together with a synchronous liver resection [7]. According to traditional surgical teaching pancreatic resection should not be contemplated when other organs are involved by the pancreatic malignancy. However, due to progresses in surgical technical expertise as well as improvements in the operative and postoperative management pancreatic surgery can be carried out with increasing safety nowadays with mortality rates of less than 5% at high volume centers [8, 9]. Therefore in other areas of pancreatic surgery such as vascular involvement of the tumor an expansion of resection criteria has been suggested with promising results in an attempt to increase the curability of pancreatic cancer [10, 11]. In this context a debate on whether to expand resection criteria in the case of incidental synchronous liver metastases appears to be contemporary. Of course various complications such as bile leak, hemorrhage, and hepatic abscess formation have been reported after liver resection [12]. Still, although the benefits of extended resections have yet to be proven, it has been shown that at least in regard to technical feasibility multivisceral resections can safely be performed in high volume centers [13]. Several authors have reported an improved long-term survival after palliative pancreaticoduodenectomy in comparison to palliative bypass surgery in patients with advanced pancreatic adenocarcinoma [14, 15]. Studies investigating therapeutic options in the setting of synchronous liver metastases are limited though. It is clear, that such an aggressive surgical approach like an additional liver resection might only be justified by an improvement of survival and/or quality of life. Furthermore surgical related morbidity and mortality must not exceed those of palliative interventions. The aim of this retrospective study therefore was to analyze the impact of pancreatic resection and simultaneous liver-directed therapy for HMPA and to assess the impact of intraoperatively detected synchronous liver metastases with regards to the surgical results and long-term survival.

## 2. Patients and Methods

In a retrospective analysis 22 patients who underwent pancreatic resection and synchronous liver resection for hepatic metastasized pancreatic adenocarcinoma (HMPA-group) between January 1, 2004 and January 1, 2009 were identified. In a next step these 22 patients were matched to 22 patients who underwent pancreatic resection for nonmetastasized pancreatic adenocarcinoma (NMPA-group) and who were homogeneous in regards of gender, age, tumor-stage (t-stage), and type of pancreatic resection performed to achieve a matched-pair-analysis.

### 2.1. Preoperative Evaluation

Standard preoperative clinical diagnostics included physical examination and routine laboratory testing, including the tumor markers CEA and CA 19-9. Computed tomography (CT) and/or magnet resonance imaging (MRI) were routinely used as radiological diagnostic tools. Patients were excluded from resection if metastatic disease was diagnosed in the preoperative evaluation. In each case of the 22 patients, who underwent pancreatic resection and synchronous liver-directed therapy, liver metastases were not detected in the preoperative radiological workup but during operative exploration as an incidental finding. Given the fact that there are no published guidelines for such situations the decision to continue with radical surgery was made by the surgeon in a case-by-case evaluation while taking into consideration the local resectability and comorbidity.

The diagnosis of pancreatic adenocarcinoma was confirmed by histopathological examination. All patients received adjuvant chemotherapy with Gemcitabin in the postoperative course.

### 2.2. Surgical Technique

Pancreatic head resection was performed in 34 patients (77%) either as pylorus-preserving (30 patients) or classic Kausch-Whipple procedure (4 patients). Pancreatoenteral anastomosis was carried out by pancreaticogastrostomy in 22 patients (65%) and pancreaticojejunostomy in 12 patients (35%). Two patients (5%) underwent distal pancreatectomy with direct closure of the pancreatic remnant using hand sutures. Eight patients (18%) received total pancreatectomy. 

All pancreatic resections were performed by experienced visceral surgeons and accompanied by standard lymphadenectomy. In the 22 patients who received a synchronous liver-directed therapy liver segmentectomy was performed in 7 patients (32%), an enucleation of the hepatic metastases was performed in 15 patients (68%).

### 2.3. Data Collection

The following data were collected for each patient: demographics (gender, age); body-mass-index; preoperative symptoms (weight loss > 10% of body weight, jaundice, unspecific epigastric pain, performance status according to Karnofsky-index); preoperative laboratory findings such as Bilirubin, Albumin, and INR, as well as serum levels of CA 19-9 and CEA; preoperative endoscopic stenting; operative details such as operation time, intraoperative blood loss, and intraoperative administered erythrocyte concentrates; results of the final histopathological examination as margin resection status (R-status), T-stage, and lymph node status, details of the postoperative course such as postoperative morbidity (according to Clavien) in terms of pancreatic fistula formation (POPF), postpancreatectomy hemorrhage (PPH), and delayed gastric emptying which were all classified according to ISGPF-definitions [16–18]; length of hospital stay; adjuvant therapy; long-term followup was assessed by our oncological outpatient clinic and by contacting the patients or general.

### 2.4. Statistical Analysis

For the statistical analysis PASW statistics 19 (SPSS Software, IBM Company, Chicago, IL, USA) was used. Summary statistics were reported using mean or median values where appropriate. Survival analysis was performed using the Kaplan-Meier method (log-rank test). Categorical variables were described using frequencies and percent. For categorical variables chi-square tests were used. A *P* value < 0.05 was defined as significant.

## 3. Results

Between January 1, 2004 and January 1, 2009 a total of 231 consecutive pancreatic head resections were performed for pancreatic adenocarcinoma at our institution. A retrospective analysis of these consecutive patients revealed a total of 22 patients (4%) who received an additional synchronous liver-directed therapy due to hepatic metastasized pancreatic adenocarcinoma. In accordance with the matched-pair design 44 patients were therefore included in our study.

### 3.1. Demographics

The median patient age was 57.5 years (31–78 years) and the male to female ratio was 64% to 36%. Tumor-associated symptoms were present in 21 patients (95%) in both HMPA- and NMPA-group at the time of diagnosis. In the HMPA-group weight loss was observed in 9 patients (41%), jaundice in 11 patients (50%), and symptoms of epigastric pain in 13 patients (59%), whereas in the NMPA-group weight loss was present in 8 patients (36%), jaundice in 13 patients (59%), and epigastric pain in 12 patients (55%). Mean body-mass-index was 23.4 (17.7–31.2) in the HMPA-group versus 23.6 (18.7–30.5) in the NMPA-group. A Karnofsky-index < 80% was observed in 10 patients (45%) of the HMPA-group versus 7 patients (32%) in the NMPA-group (Table 1).

### 3.2. Laboratory Findings

At presentation mean serum-level of CA 19-9 was 8427.6 ku/L (±25812.9) and mean serum level of CEA was 14.3 ug/L (±17.5) in the HMPA-group versus CA 19-9 of 1019.9 ku/L (±1350.8) and CEA of 5.8 ug/L (±6.3) in the NMPA-group. The differences in serum tumormarker levels were not significant (*P* value = 0.322 and *P* value = 0.116) between the two groups. Mean laboratory values of bilirubin (5.0 mg/dL versus 4.7 mg/dL), Quick (103.7 TPZ versus 97.2 TPZ), and albumin (4.2 IU versus 3.7 IU) also did not statistically differ between the two groups. An endoscopic stent therapy prior to surgery was performed in 15 patients (68%) in both groups (Table 1).

### 3.3. Operative Details

Mean operation time was 330.2 minutes (±80.9) and median intraoperative blood loss was 750 mL (±345.7) in the HMPA-group versus 349.3 minutes (±56.1) and 700 mL (±767.0) in the NMPA-group. Intraoperative administration of Erythrocyte-concentrates was necessary in 8 patients (36%) of the HMPA-group and in 9 patients (41%) of the NMPA-group. These differences however were not significant between the two groups (*P* value = 0.243, *P* value = 0.333) (Table 2).

### 3.4. Postoperative Outcome

In the postoperative course surgical complications (≥Clavien 3) occurred in 4 patients (18%) of the HMPA-group and 9 patients (41%) of the NMPA-group. In the HMPA-group POPF occurred in 2 patients (9%)—both POPF grade C—and PPH in 2 patients (9%)—PPH grade A and grade C in one patient each. In the NMPA-group surgical complications were observed in 9 patients (41%). POPF grade C occurred in 2 patients (9%) and PPH grade C in 1 patient (5%). DGE was observed in 1 patient (5%) within the NMPA-group. There were more postoperative complications (≥Clavien 3) in the NMPA-group. These differences however reached no significance (*P* value = 0.099). The incidence of POPF (*P* value = 1), PPH (*P* value = 0.550) and DGE (*P* value = 0.312) did not significantly differ between the two groups. Revision surgery was performed in 2 patients (9%) of the HMPA-group and 4 patients (18%) of the NMPA-group, this comprised two residual pancreatectomies and two Sewing-over of the panreatoenteral anastomosis each due to grade C POPF, one insufficiency of the biliary-enteric anastomosis, and one instance of postoperative hemorrhage. Mean length of hospital stay was 23.3 days in the HMPA-group versus 23.9 days in the NMPA-group (*P* value = 0.893) (Table 3). No perioperative mortality occurred in either one of the groups.

### 3.5. Histopathological Findings

With regard to surgical radicality a margin negative resection status (R0-status) was reached in 7 patients (32%) in the HMPA-group versus 13 patients (59%) in the NMPA-group. Positive lymph node status (pN1) was found in 18 patients (82%) in the HMPA-group in comparison to 20 patients (91%) in the NHPA-group. Tumor stages were pT1 in 1 patient (5%), pT3 in 17 patients (77%), and pT4 in 4 patients (18%) in both groups (Table 2).

### 3.6. Survival Analysis

**Figure 1:**
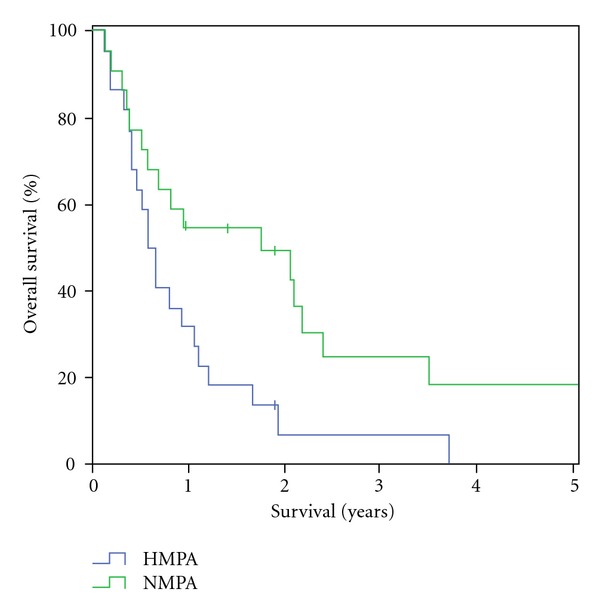
Patient overall survival: HMPA versus NMPA.

**Figure 2:**
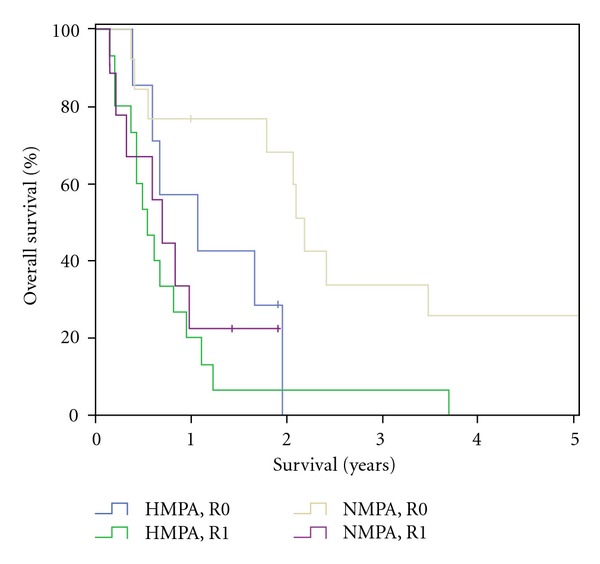
Patient survival in regards of surgical radicality (R0, R1): HMPA versus NMPA.

**Figure 3:**
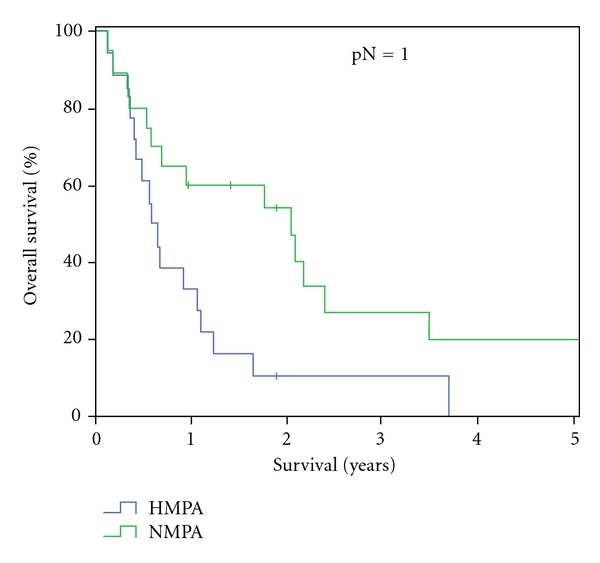
Patient survival in regards of positive lymph node status (pN1): HMPA versus NMPA.

The overall median survival in the HMPA-group was 228 days (±298.0), with a two-year survival of 5% (one patient). No five-year survival was reached within this group. In the NMPA-group median survival was 437 days (±681.8) and therefore significantly longer than in the HMPA group (*P* = 0.15). Within the NMPA-group a two-year survival was reached in 8 patients (36%) and a five-year survival in 3 patients (14%) (Figure 1). Median survival for R0 resected patients was 390 days (±195.1) in the HMPA group versus 794 days (±33.5) in NMPA group. In patients with R1 resections median survival was 194 days (±40.6) in the HMPA group and 255 days (±61.2) in the NMPA group (Figure 2). With positive lymph node involvement (pN1) median survival was 215 days (±30.8) in the HMPA group versus 754 days (±249.2) (Figure 3).

## 4. Discussion

Pancreatic surgery is considered the gold standard treatment of locally resectable and nonmetastasized pancreatic adenocarcinoma and can nowadays be carried out with mortality rates of less than 5% at high volume centers even in an advanced stage of disease [8]. Resection criteria have not been clearly defined though and therefore remain to be debated. In the past, patients with locally advanced disease, vascular involvement (hepatic artery, superior mesenteric artery, or the superior mesenteric vein/portal vein axis) or metastases were traditionally referred to conservative palliative treatment approaches which include a wide range of medical, surgical, and other interventions [19]. However, in recent randomized prospective trials and meta-analysis patients benefit of classic palliative procedures with regard to quality of life and survival have been questioned [20, 21]. With improving safety and surgical expertise several authors have suggested more aggressive, curative-intended approaches in pancreatic surgery to improve long-term survival even in patients with advanced pancreatic adenocarcinoma [22]. Additional vascular resections, for example, are nowadays considered a standard procedure with good results in regards of perioperative and long-term outcome [23, 24]. In our study, all patients of the HMPA-group had incidental liver metastases identified during exploration at laparotomy—a not unusual finding in pancreatic surgery. An explanation for this setting may be that on one hand the accuracy of detecting liver metastases even with high-quality CT or MR imaging in the preoperative evaluation is still limited nowadays. On the other hand time also may have elapsed between imaging and surgical exploration. In the context of HMPA, surgery—whether curative or palliative—is still discussed controversially. In other fields of oncological surgery, most commonly in colorectal cancers or neuroendocrine tumors, but also in nontraditional tumors as sarcoma, melanoma, and squamous cell carcinoma hepatic resection of metastases provide a clear survival benefit [14, 25–28]. Experience with liver resection of hepatic metastases from pancreatic adenocarcinoma is limited to a few patients only though. Adam et al. reported 5-year survival rates of up to 25% for patients who underwent hepatic resection of metastatic lesions from pancreatobiliary primary tumors [29]. A great majority of the included patients had metachronous metastases though and underwent a staged procedure after a prognostic-positive disease-free interval. The results of this study can therefore only hardly be alienated on the intraoperative assessment on whether or not to perform simultaneous liver resection in HMPA. Klempnauer et al. reported one-year survival rates of 41% after synchronous and 40% after metachronous resection of solitary liver metastases in patients with HMPA [30]. Shrikhande et al. compared the outcome in patients with HMPA after synchronous liver-directed therapy to patients who underwent exploratory laparotomy with or without palliative bypass and reported a median survival of 11.4 months in the patients who underwent synchronous liver resections as opposed to 5.9 months in the patient group who underwent primary palliative surgery [31]. According to these results simultaneous liver resection may seem reasonable also as a palliative approach. Other authors on the other hand strongly recommend against performing extended surgical treatment approaches in the event of synchronous liver metastases [32]. Takada et al., for example reported their experience with resection of periampullary or pancreatic adenocarcinomas with synchronous hepatic metastases and found that not only overall survival was not improved but also that surgical morbidity and mortality were increased with additional liver-directed therapy [33]. These results are underlined by the study of Elias et al. who had no one-year survivors after simultaneous hepatic resection with pancreaticoduodenectomy in patients with HMPA [34]. Generally, as opposed to, for example, colorectal surgery the primary required surgical procedure in the treatment of pancreatic adenocarcinoma itself—most commonly a pancreaticoduodenectomy—is associated with significant morbidity and eventually mortality. A synchronous liver resection carries the additional risk of bile leak, hemorrhage, and hepatic abscess and may therefore contribute to an increased risk for postoperative morbidity [12]. An increased risk of developing a liver abscess as an example has been reported with 40% to 50% for simultaneous liver resections and may be explained by the construction of a biliary-enteric anastomosis during pancreaticoduodenectomy [34]. Kamphues et al. also showed that postoperative complications deteriorate long-term outcome in pancreatic cancer patients [35]. An extended pancreas resection must therefore be well considered. The results of our study account that pancreas resection and synchronous liver-directed therapy may be carried out safely for HMPA with a postoperative morbidity of 18%. No “liver-specific” complications were observed. Also operative factors such as operation time, intraoperative blood loss or the amount of intraoperative administered erythrocyte concentrates were not significantly increased in comparison to pancreas resections alone in NMPA-patients. In accordance to recent studies the results of our study demonstrate that surgical therapy of HMPA is not limited by technical feasibility and not automatically associated with an increase in postoperative complications [36]. A margin negative resection status (R0-status) was at least reached in 7 patients (32%) in the HMPA-group. As expected, overall survival was significantly decreased in the presence of liver metastases. However, 1-year survival was achieved in 7 patients (32%) in the HMPA-group with 4 patients (18%) exceeding an overall-survival >500 days. It may therefore be concluded that individual patients indeed benefit from a radical resection in the setting of HMPA. Of course the presence of liver metastases has a negative impact on overall-prognosis. The question is rather should all patients with the constellation of HMPA automatically be referred to standard palliative bypass surgery or primary chemotherapy, especially in the event of a suspected early dissemination stage as in the finding of incidental liver metastases which were not detected in the preoperative diagnostic. An additional aspect in this context may also be that occult liver metastases may already be present in parts of patients with pancreatic adenocarcinoma even if not detected during operative exploration especially in the presence of portal vein infiltration. As a conclusion the results of our study underline the results of Klempnauer et al. who concluded that the prognosis of patients with HMPA should not uniformly considered to be hopeless [30]. The question on whether patients with HMPA generally benefit from a radical surgical approach, especially in view of a better life quality and/or improved overall survival can of course only hardly be answered using this study constellation. Further studies would have to compare this radical surgical approach to patients who either underwent classic palliative bypass-surgery or primary chemotherapy in order to possibly identify general advantages especially with regard to life quality and overall survival. In the context of continuous progress in peri- and postoperative management which lead to a decrease in operation associated morbidity and mortality in the last years, we should continuously reevaluate our data with regard to overall survival and as a conclusion therefore eventually adjust our guidelines in the interdisciplinary oncologic therapy management. As mentioned, this question cannot be answered by our study. We can only constellate that simultaneous pancreas and liver resection is a safe and technical feasibly approach and that individual patients possibly benefit from this procedure. In the event of an increased operative risk score and/or locally advanced tumor stage—in accordance to recent literature—patients should still be referred to the classic palliative therapeutic approaches. Further studies are necessary though to investigate a possible general benefit—if only by a tumordebulking—of an extended pancreas- and liver resection in comparison to the standard classic palliative approaches. The statistical power of our study is surely limited by the relatively small sample size of patients. Also, the retrospective study design may have lead to a selection bias in how patients were chosen for simultaneous liver directed therapy. All patients in our study underwent synchronous resection of the primary pancreatic tumor and the liver metastases—an approach (as opposed to the staged procedure) not just to avoid complications caused by preliminary surgery but also to “win time” in the balance between curative surgery and further adjuvant therapy. De Jong et al. also reported that patients with staged liver-directed therapy were significantly more likely to develop a liver abscess compared with patients who underwent a simultaneous pancreaticoduodenectomy and liver-directed therapy [37]. Patients with HMPA considered for extended surgery need to be carefully selected by evaluating patients age, general condition and also assessing the complexity of the primary radical resection of the pancreatic tumor. Furthermore the extent of the liver resection needs to be taken into plausible consideration. In our study 15 patients (68%) underwent an enucleation of solitary liver metastases, a segmentectomy was performed in 7 (32%) patients. More extensive liver resections such as left or right hemihepatectomy were avoided and need to be evaluated carefully. To avoid the finding of advanced tumor stages during laparotomy some authors have advocated the routine use of diagnostic laparscopy before laparotomy in patients with a preoperative risk constellation such as preoperatively elevated CA 19-9 levels >300 U/L [38]. Besides all surgical approaches adjuvant chemotherapy remains essential [39]. In the future, more patients will eventually qualify for radical surgery even with an advanced stage of pancreatic adenocarcinoma. With further medical progress the borders between curative and palliative surgical approaches need to undergo constant reevaluation with regard to their impact on overall survival and/or quality of life.

## 5. Conclusion

Simultaneous pancreas resection and liver-directed therapy is a complex procedure but may be carried out safely at high-volume centres nowadays with promising survival rates in individual patients after radical resection of HMPA. This approach may therefore be considered in selected patients. However, further studies will need to be carried out to identify a possible general benefit of an extended pancreas resection for patients with HMPA in view of life quality and/or overall survival in comparison to patients who undergo classic palliative bypass surgery or primary chemotherapy.

## Figures and Tables

**Table 1:** Patients characteristics.

	Pancreatic adenocarcinoma + incidental liver metastases	Nonmetastasized pancreatic adenocarcinoma	*P* value
Number of patients	22	22	
Mean age (years/range)	57.5 (31–78)	57.5 (42–74)	
Gender (male)	14 (64%)	14 (64%)	
Tumor-associated symptoms			
Weight loss	9 (41%)	8 (36%)	
Jaundice	11 (50%)	13 (59%)	
Epigastric pain	13 (59%)	12 (55%)	
Mean body-mass-index (range)	23.4 (17.7–31.2)	23.6 (18.7–30.5)	
Mean CA 19-9 (ku/L)	8427,6	1019,9	0.322
Mean CEA (ug/L)	14.3	5.8	0.116
Mean bilirubin (mg/dL)	5.0	4.7	0.911
Mean quick (TPZ)	103.7	97.2	0.125
Mean albumin (IU)	4.2	3.7	0.113
Preoperative endoscoping stenting	15 (68%)	15 (68%)	

**Table 2:** Operative course and histopathological findings.

	Pancreatic adenocarcinoma + incidental liver metastases	Nonmetastasized pancreatic adenocarcinoma	*P* value
Pylorus preserving pancreaticoduodenectomy	16 (73%)	14 (64%)	
Whipple procedure	1 (5%)	3 (14%)	
Total pancreatectomy	4 (18%)	4 (18%)	
Distal pancreatectomy	1 (5%)	1 (5%)	
Liver directed therapy	22	0	
Enucleation	15 (68%)	0	
Segmentectomy	7 (32%)	0	
Mean operation time (minutes)	330.2	349.3	0.243
Median intraoperative blood loss (mL)	750	700	0.333
Surgical radicality			
R0	7 (32%)	13 (59%)	
R1	10 (46%)	7 (32%)	
R2	5 (23%)	2 (9%)	
Positive lymph node status (pN1)	18 (82%)	20 (91%)	
T-Stage			
pT2	1 (5%)	1 (5%)	
pT3	17 (77%)	17 (77%)	
pT4	4 (18%)	4 (18%)	

**Table 3:** Postoperative course.

	Pancreatic adenocarcinoma + incidental liver metastasis	Nonmetastized pancreatic adenocarcinoma	*P* value
Surgical complications (Clavien grade ≥ 3)	4 (18%)	9 (41%)	**0.099**
Postoperative pancreatic fistula (POPF)	2 (9%)	2 (9%)	1
Postpancreatectomy hemorrhage (PPH)	2 (9%)	1 (5%)	0.550
Delayed gastric emptying (DGE)	0	1 (5%)	0.312
Reoperations	2 (9%)	4 (18%)	0.216
Mean length of hospital stay (days)	23.3	23.9	0.893
Perioperative letality	0	0	
